# Efficacy of Primate Humoral Passive Transfer in a Murine Model of Pneumonic Plague Is Mouse Strain-Dependent

**DOI:** 10.1155/2014/807564

**Published:** 2014-07-06

**Authors:** V. A. Graham, G. J. Hatch, K. R. Bewley, K. Steeds, A. Lansley, S. R. Bate, S. G. P. Funnell

**Affiliations:** Microbiological Services, Public Health England, Porton Down, Salisbury, Wiltshire SP4 0JG, UK

## Abstract

New vaccines against biodefense-related and emerging pathogens are being prepared for licensure using the US Federal Drug Administration's “Animal Rule.” This allows licensure of drugs and vaccines using protection data generated in animal models. A new acellular plague vaccine composed of two separate recombinant proteins (rF1 and rV) has been developed and assessed for immunogenicity in humans. Using serum obtained from human volunteers immunised with various doses of this vaccine and from immunised cynomolgus macaques, we assessed the pharmacokinetic properties of human and cynomolgus macaque IgG in BALB/c and the NIH Swiss derived Hsd:NIHS mice, respectively. Using human and cynomolgus macaque serum with known ELISA antibody titres against both vaccine components, we have shown that passive immunisation of human and nonhuman primate serum provides a reproducible delay in median time to death in mice exposed to a lethal aerosol of plague. In addition, we have shown that Hsd:NIHS mice are a better model for humoral passive transfer studies than BALB/c mice.

## 1. Introduction

Plague is caused by the gram-negative bacterium* Yersinia pestis*; it is primarily a disease of rodents, which can cause zoonotic disease directly via the aerosol route or indirectly via arthropod vectors. Throughout history, human pandemics have been caused, the greatest being during the Middle Ages where at least 25% of the population was killed [1]. There is a general perception that plague is nonexistent; however, there are several regions in the world where plague is still endemic and there is a consistent annual morbidity and mortality rate with the potential to cause large outbreaks [2]. Worldwide, from 2000 to 2009, at least 21,725 people were infected with plague and 1,612 people died, including 7 deaths in the United States [3].

Humans are extremely susceptible to plague and the aetiology of the disease is dependent upon the route and source of the infection, resulting in one of the three principal clinical forms: bubonic, septicaemic, and pneumonic plague [2]. Pneumonic plague follows inhalation of aerosolised droplets containing* Y. pestis.* It has the highest fatality rate of the three forms of plague, with a 1–3 day incubation period. It has a 100% fatality rate unless antibiotics are given the same day as symptoms develop [3]. Once inhaled the plague bacteria multiply in the alveolar spaces, and the patient is usually infectious 1-2 days after infection; during this time the patient produces highly contagious aerosolised* Y. pestis* in fine droplets, which can be inhaled deep into the respiratory tract of close contacts [2].

Antibiotics have been used for the treatment of plague and, to date, there have been two reports of antibiotic resistant plague strains [4, 5]. Laboratory experiments have shown that it is possible for* Y. pestis* to acquire plasmids which contain antibiotic resistant genes [3, 5]. Due to the rapid onset and the high case fatality rate of pneumonic plague, the potential bioterrorist threat, and the potential emergence of antibiotic resistant strains, the production of a vaccine to enable protection from this form of plague is required.

Vaccines against plague have previously been limited to live-attenuated or formalin-killed whole cell* Y. pestis*. They have not shown protection against primary pneumonic plague and have shown adverse reactions to the vaccine itself [6, 7]. Therefore, there is a requirement for vaccines which will provide long-lasting protection against all forms of plague infection with minimal side-effects.

It has been shown that both humoral and cellular immunity are required for complete plague immunity [8–12]; however, antibodies against two natural virulence proteins F1 and V are associated with protection during a natural infection and have been shown to be protective in mouse models of pneumonic plague when given as recombinant proteins [11, 13–15]. The F1 capsular protein is unique to* Y. pestis* and is an antiphagocytic protein capsule, the gene for which is located on the pFra plasmid. The V protein is an outer membrane protein encoded by the pYV plasmid and is part of the Type III secretion system [3, 8, 11]. These (rF1 and rV) are the major constituents of new subunit plague vaccines [12, 16–24].

Due to the lack of an endemic population within which new plague vaccines could be assessed the FDA will allow licensure based on the animal rule (21CFR 601.91 Subpart H). There are several critical components in evaluating vaccine efficacy under this rule. Licensure will require the use of an assay(s) that measures a functional component of the immune response and is reasonably likely to predict clinical benefit. Scientists and regulatory authorities have for many years been looking for a functional assay which will enable measurement of a correlate of protection against pneumonic plague. Passive immune protection studies in animals, using antibodies isolated from vaccinated individuals, may provide this assay [14, 15, 25, 26].

The experiments described here detail the development of a pneumonic plague mouse model and the subsequent use of this model to test the* Y. pestis* subunit vaccine containing recombinant F1 and recombinant V (rF1 and rV) by the passive transfer of unfractionated serum and plasma from immunised cynomolgus macaques and humans.

This is the first paper to assess the ability of the rF1 and rV vaccine to generate protection from an aerosol challenge of the CO92 strain of* Y. pestis* by passive transfer of unfractionated serum. Previous papers have assessed the combined rF1V fusion protein [13]. Known titres of anti-rF1 and anti-rV antibodies were then transferred into groups of immunologically naïve mice to assess the ability of humoral immunity to protect against pneumonic plague.

## 2. Materials and Methods

### 2.1. Bacterial Strain

*Y. pestis* strain CO92 (biovar Orientalis, NR641, BEI Repositories) was supplied by the Biodefence and Emerging Infections (BEI) Research Repository (USA) in accordance with International Export and Import Regulatory Requirements. The organism was stored and handled in accordance with US Biological Select Agent or Toxin requirements.

### 2.2. Bacterial Growth and Subculture

The generation of the master stock was performed by streaking* Y. pestis* onto tryptic soy agar (TSA) (VWR, UK) and incubated at 26°C for 48 hours. This was used to inoculate tryptone soya broth (TSB) (Media Services, PHE) which was incubated overnight at 26°C. This broth was then used to inoculate a further suspension of TSB, which was incubated at 26°C overnight. A 50% glycerol (Sigma, UK) solution was added to the broth to a final concentration of 40% (v/v) glycerol. The master stocks were frozen at −80°C. Working stocks were generated from a vial of master stock. This was performed by streaking the master stock of* Y. pestis* onto TSA and incubating at 26°C for 72 hours. A strike through of the lawn was used to inoculate a large volume of TSB and was incubated overnight at 26°C. A 50% glycerol solution was added to the broth to a final concentration of 40% (v/v) glycerol. The working stocks were frozen at −80°C. Master stock certificate of analysis was produced; characterisation of these stocks included microscopic staining (Wayson's), colony morphology and purity checks on TSA, Congo red uptake on Congo red agar, multiple loci (5 target) PCR, 16S rRNA sequencing, and VNTR for genetic integrity checks. Subcutaneous lethality checks were performed on BALB/c mice and stocks were 100% lethal (data not shown). All experimental and confirmatory studies were performed using the working stock vials; therefore they had a maximum passage number of 3.

### 2.3. Preparation of Inoculum

All inocula were prepared in the same way. A vial from the working stock was thawed at ambient temperature and then streaked onto TSA and incubated at 26°C for 54 hours for subculture and purity check. The contents of individual streak plates were washed into 25 mL total of TSB. To obtain maximal yields, the broth was incubated with orbital agitation at 26°C for 18 hours. Following incubation, 25 mL fresh TSB was added and the optical density was measured at 600 nm wavelength. The broths were then reincubated with orbital agitation for 3 hours at 26°C. The broths were harvested when the OD_600 nm_ values were over 3, with an aliquot taken for real time PCR analysis.

### 2.4. Determination of Bacterial Challenge Dose

Retrospective quantification of colony forming unit concentration of inocula was conducted using a spread plate method. Briefly, serial ten-fold dilutions were created to bring the bacterial suspensions into a countable range. In duplicate, 100 *μ*L of each diluted bacterial suspension was spread across the surface of a TSA plate. After 48 hours incubation, the plates were manually counted for colony forming unit content and the average counts used to determine colony forming unit concentration (CFU/mL). Immediately prior to aerosol exposure of mice all inocula were checked for the presence of plasmids and essential genetic characteristics by PCR, as described below.* In vivo* studies were not initiated without confirmation of genetic integrity by PCR for each batch of inocula culture.

### 2.5. Confirmation of Strain Virulence

A four target real time PCR was utilised to ensure genetic integrity and stability for each batch of inocula used for* in vivo* studies. This confirmed the presence of all three plasmids and the* pgm* virulence locus [27] of the bacterial chromosome. The PCR was based on the targets detailed in Table 1. PCR reaction constituents consist of 2× fast universal master mix (Applied Biosystems) 10 *μ*L; forward primer (900 nM); reverse primer (900 nM); probe (250 nM); DNA template 2 *μ*L; and nuclease-free water to a final volume of 20 *μ*L. PCR thermal cycling consisted of 40 cycles (ABI 7500 fast protocol) comprising 95°C, 3 seconds, and 60°C, 30 seconds.

### 2.6. Mice

In accordance with UK Home Office regulations, BALB/c and Hsd:NIHS mice were obtained from UK accredited suppliers (Harlan, UK, or Charles River, UK). In all studies described, mice were required to comply with both age and weight selective criteria: age minimum 8–10 weeks and a minimum body weight of 17 grams. Mice were randomly assigned and housed in groups of between five and ten. Food and water available to all mic* ad libitum*, and were exposed to 12 hour. Pilot studies were performed with groups of 5 or 6 mice. Details for all the challenge experiments can be found in Table 2. All animal procedures were conducted under the authority of and in accordance with a UK Home Office license.

### 2.7. Health Monitoring

For three days before infection and throughout the remainder of the studies, mice were weighed in the morning and afternoon to monitor diurnal body-weight fluctuation. In addition, the health status of each mouse was checked on at least two occasions every day. When mice began to show signs of infection, the monitoring frequency was increased to at least four occasions every day. When mice were seen to have restricted movement and reflexes they were humanely euthanised using a UK Home Office schedule 1 procedure.

### 2.8. Human Serum

All human serum samples were from individuals vaccinated with either 40, 80, or 120 *μ*g of recombinant plague vaccine using the same dosing regimen and serum isolated 35, 196, or 365 days postvaccination. All human serum samples were provided by NIAID (through Avecia and PharmAthene) under contract number N01-AI30062.

### 2.9. Pharmacokinetic Analysis of Human Antibodies

Pooled human serum with known ELISA titres against rF1 and rV proteins was injected into four groups of five BALB/c mice via the intraperitoneal route (250 *μ*L/mouse). Groups of mice were serially sacrificed at 1.5, 3, 6, and 12 hours after injection. One group of 5 mice was injected with 250 *μ*L/mouse negative control serum for a control. The levels of circulating human IgG antibody levels specific for rF1 and rV were determined by ELISA (performed by Huntingdon Life Sciences, UK).

### 2.10. Passive Humoral Therapy of Human Serum

Three to six hours before aerosol infection, groups of five mice were administered with 250 *μ*L of human serum via the intraperi-toneal route. Mice were administered with either test, positive control or negative control human serum. The mice were then transferred into high-containment isolators before being loaded into restraint tubes for aerosol administration.

### 2.11. Aerosol Challenge of Mice

Groups of mice were challenged for 10 minutes with a dynamic aerosol of* Y. pestis* (<2 *μ*m, mass median aerodynamic diameter) using a contained modified Henderson apparatus [28]. The challenge aerosol was generated using a three-jet Collison nebulizer (BGI, Waltham, USA) containing 20 mL* Y. pestis* in TSB. The resulting aerosol was mixed with conditioned air (65% relative humidity, 22°C) in the spray tube and delivered to the nose of each animal via an exposure tube in which the unanaesthetised mice are held in restraint tubes. Samples of the aerosol were collected into 20 mL TSB using an SKC Biosampler (SKC, Dorset, UK) operating at 12.5 L/min. In the later studies using the Hsd:NIHS mice, aerosol samples were collected using an All Glass Impinger (AGI30, Ace Glass, USA) [29] operating at 6 L/min and an Aerodynamic Particle Sizer (TSI Instruments Ltd., Bucks, UK), controlled using the AeroMP management platform (Biaera Technologies LLC., MD, USA). Counts were used to calculate the inhaled dose using a derived respiratory minute volume estimated from the average weight of the animals [30].

### 2.12. ELISA

Recombinant F protein (rF1) and V protein (rV) (provided by Pharmathene via NIAID) were coated in carbonate coating buffer separately into wells of 96-well microplate (Nunc Maxisorp C96, Fisher Scientific, UK) and incubated overnight at 4°C. The plates were washed in PBS containing 0.05% w/v Tween-20 (Sigma, UK) before and after each absorptive reagent addition. Plates were blocked for 2 hours with PBS containing 3% w/v bovine serum albumin (BSA) (Sigma, UK). Where necessary, samples were prediluted using PBS containing 1% w/v BSA and 0.05% Tween-20. Murine samples containing human antibody were incubated for 1 hour, and vaccinated-goat serum was used as a positive control (rF1 14182 Pro-Sci, USA; rV 14181 Pro-Sci, USA). Bound antibody was detected with horseradish peroxidase conjugated to protein-G (Pierce, USA). Bound conjugate was detected by addition of ABTS peroxidase substrate system (Kirkegaard and Perry Laboratories, USA), and the reaction stopped with ABTS peroxidase stop solution (Kirkegaard and Perry Laboratories, USA). Plates were read at a wavelength of 405 nm using an automated ELISA reader (VersaMax, Molecular Devices, CA, USA) and data was analysed using Softmax Pro version 4.7.1. (Molecular Devices, CA, USA). The goat positive control sera (anti-rF1 or anti-rV) were assigned arbitrary values of 10000 units per mL. The human and cynomolgus macaque sera titres were extrapolated from the goat positive control sera.

### 2.13. Cynomolgus Macaques (*Macaca fascicularis*)

Two female cynomolgus macaques of Mauritian origin were obtained from a UK breeding colony for use in this study. Both animals weighed more than 2.5 kg and were over 2 years of age at immunization. Animals were housed according to the Home Office (UK) Code of Practice for the Housing and Care of Animals Used in Scientific Procedures (1989) and the National Committee for Refinement, Reduction and Replacement (NC3Rs) Guidelines on Primate Accommodation, Care and Use, August 2006. When a procedure required the removal of a primate from a cage it was sedated by intramuscular (*i.m*.) injection with ketamine hydrochloride (10 mg/kg) (Ketaset, Fort Dodge Animal Health Ltd., Southampton, UK). All procedures were conducted under a project license approved by the Ethical Review Process of Public Health England, Salisbury, UK, and the UK Home Office. None of the animals had previously been used for experimental procedures.

### 2.14. Generation of Cynomolgus Macaque Immune Serum

Two cynomolgus macaques were immunized then twice boosted with a recombinant plague vaccine consisting of rF1 and rV antigens with an alhydrogel adjuvant (plague (recombinant) vaccine suspension, 873082-H01, Batch 7051, Avecia Biologics Ltd., UK). A 20 ug/mL solution was prepared by adding 0.25 mL of the vaccine (which contained 240 ug/mL of each antigen adjuvanted with 0.26% (w/w) alhydrogel) to 2.75 mL 0.26% (w/w) alhydrogel in a sterile vessel and mixed thoroughly. The vaccination schedule comprised of three intramuscular injections of 10 *μ*g of total antigen with a 21 day interval between injections.

### 2.15. Pharmacokinetic Analysis of Cynomolgus Macaque Antibodies

Pooled cynomolgus macaque serum with known ELISA titres against rF1 and rV proteins was injected into groups of five Hsd:NIHS mice via the intraperitoneal route (250 *μ*L/animal). Groups of five mice were serially sacrificed, preinjection or at 1.5, 3, 6, and 12 hours postinjection. The levels of circulating cynomolgus macaque IgG antibody were determined by ELISA as previously described.

### 2.16. Passive Humoral Therapy of Cynomolgus Macaque Serum

Three to six hours before aerosol infection, groups of five mice were administered with 250 *μ*L/mouse of test cynomolgus macaque serum, positive control vaccinated human serum, or as a negative control nonvaccinated human serum via the intraperitoneal route. The mice were then transferred into high containment isolators before being loaded into restraint tubes for aerosol administration.

### 2.17. Statistical Analysis

Survival analysis was performed using Kaplan-Meier survival curves and comparisons were performed using Wilcoxon rank sum for *P* values [31].

## 3. Results 

### 3.1. Aerosol BALB/c Murine Infection Model

An aerosol model of plague was developed using BALB/c mice to enable assessment of the efficacy of passive transfer of antibodies against pneumonic plague. An exposure to 2.4 LD_50_ aerosolised* Y. pestis* CO92 or above was found to produce a mean time to death of 4.2 days (±0.5 SE).

The presented dose used in a range of humoral passive transfer experiments was extremely consistent and only varied between 10.6 and 13.9 LD_50_s.

### 3.2. Passive Protection of Human Antibody within BALB/c Mice

**Figure 1:**
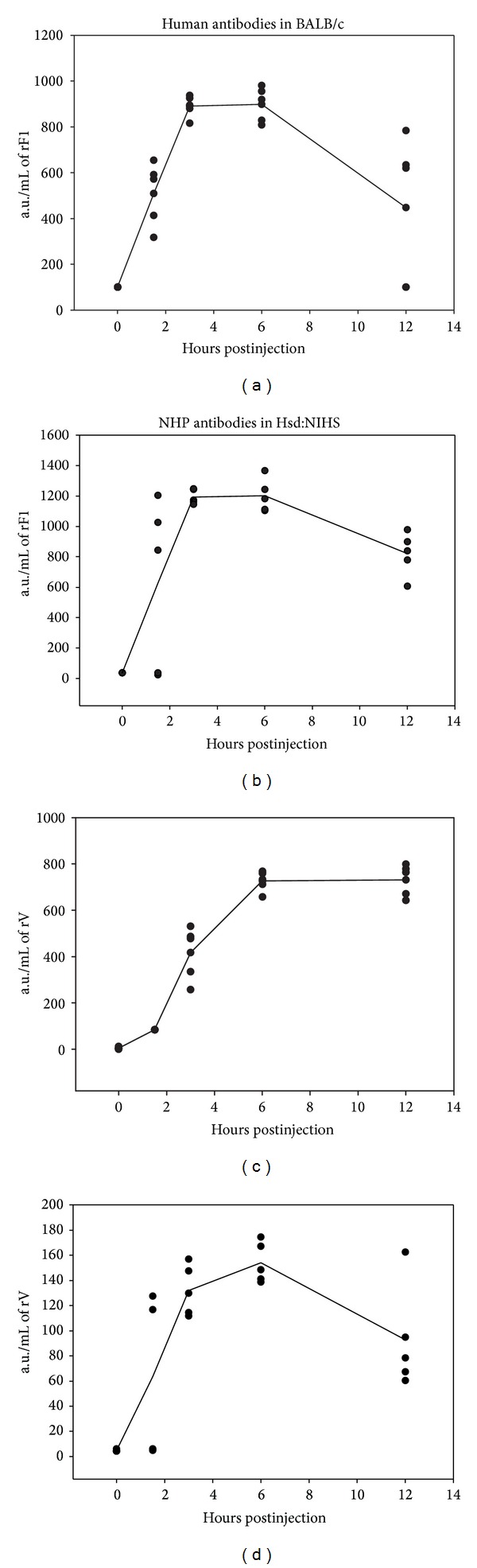
Pharmacokinetics of human or cynomolgus macaque antibody in BALB/c and Hsd:NIHS mice, respectively. 0.25 mL human ((a) and (c)) or cynomolgus macaque ((b) and (d)) plague vaccine serum was injected via the intraperitoneal route into BALB/c (*n* = 20) or Hsd:NIHS (*n* = 20) mice, respectively. The mice were serially sacrificed and the presence of anti-rF1 ((a) and (b)) or anti-rV ((c) and (d)) antibody was assessed by ELISA. Additional groups of mice (*n* = 5) were sacrificed preinjection to provide baseline data. The data suggests that the time delay between passive antibody administration and optimal serum concentration in the murine serum was between 3 and 6 hours. a.u., arbitrary units.

To maximise the efficacy of passively transferred antibodies, we examined the pharmacokinetics of human antibody in the circulating blood stream of BALB/c mice (Figure 1). The results indicated that optimal human antibody concentrations were reached between three and six hours after intraperitoneal administration into mice.

**Figure 2:**
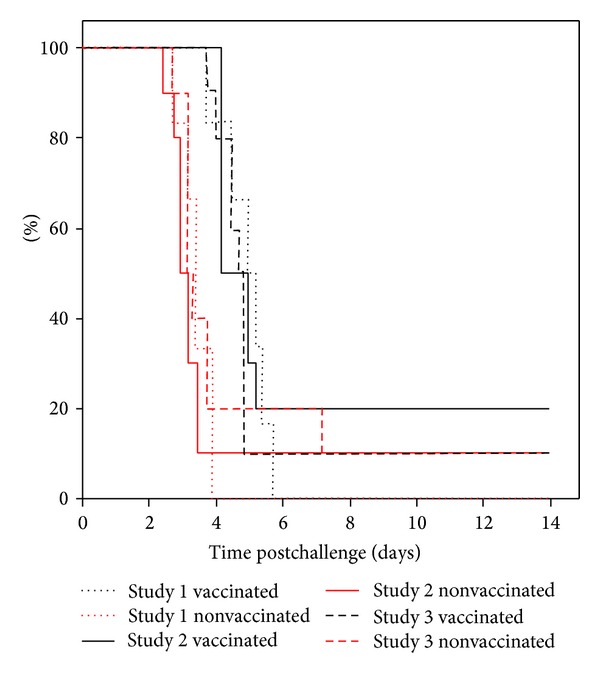
Combined survival plots of 3 studies following passive therapy of mice with either nonimmune or plague vaccinated volunteer serum. Study (1), *n* = 6 per group, study (2), *n* = 10 per group, and study (3), *n* = 10 per group. Mice were administered 250 *μ*L human serum into the peritoneal cavity, 3 hours before being exposed to more than 10 LD_50_ aerosolised* Y. pestis* using a modified contained Henderson apparatus. The median time to death (MTD) was calculated using a Kaplan-Meier survival plot.

A pool of high titre serum obtained from human volunteers that had been vaccinated with experimental recombinant plague vaccine was initially used to assess the concept that passive human antibody therapy would provide some form of protection against pneumonic plague in BALB/c mice. The combined Kaplan-Meier survival curves of three studies are presented in Figure 2. All BALB/c mice were infected three hours after intraperitoneal administration of 250 *μ*L of either nonimmune human serum or a pool of serum from vaccinated volunteers. The serum had a significant protective effect (*P* < 0.001, Wilcoxon test), as defined by a delay of the median time to death (MTD) of 1 to 2 days (Table 3).

### 3.3. Assessment of Vaccine Dose and Longevity of Protection

**Figure 3:**
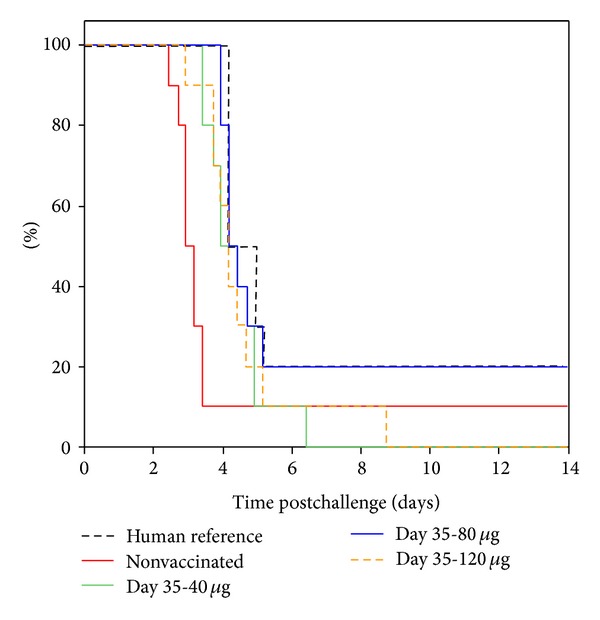
Combined survival plots of BALB/c mice following passive transfer of human serum from vaccines 35 days after receiving different doses of vaccine. Study (2), *n* = 10 per group. Mice were administered 250 *μ*L human serum into the peritoneal cavity, 3 hours before being exposed to more than 10 LD_50_ aerosolised* Y. pestis* using a modified contained Henderson apparatus. The median time to death (MTD) was calculated using a Kaplan-Meier survival plot.

The dose of the vaccine required to provide protection was investigated by examining the protective effect of human sera taken from vaccines 35 days after they received one of three different doses (40 *μ*g, 80 *μ*g, and 120 *μ*g). The sera showed a significant protective effect (*P* < 0.001, Wilcoxon test) in survival analysis (Figure 3) as defined by a delay of the MTD from 2.94 days to between 3.92 and 4.18 days (Table 3). However, there was no significant difference (*P* > 0.02, Wilcoxon test) between the three doses of vaccine.

**Figure 4:**
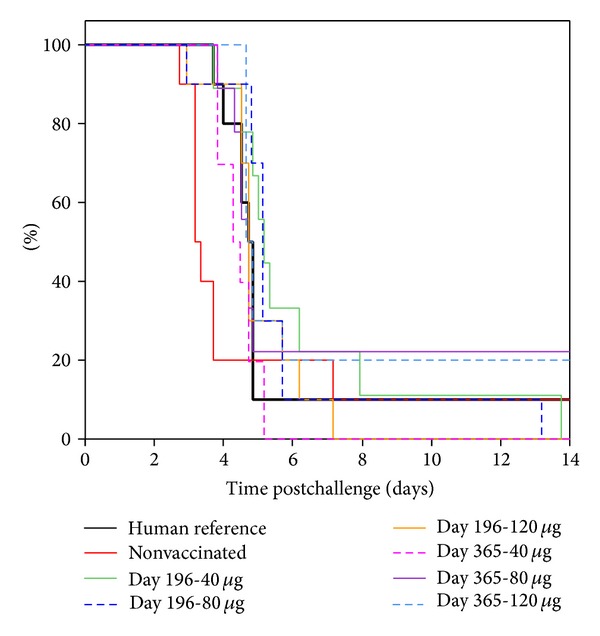
Combined survival plots of mice following passive therapy of either nonimmune or plague vaccinated volunteer serum from 196 to 365 days postinoculation. Study (3), *n* = 10 per group. BALB/c mice were administered 250 *μ*L human serum into the peritoneal cavity, 3 hours before being exposed to* Y. pestis* using a modified contained Henderson apparatus. The median time to death (MTD) was calculated using a Kaplan-Meier survival plot.

The length of the protective effect of immunisation was also tested by comparing the passive protective effect of human vaccine sera taken at 35, 196, and 365 days postimmunisation in the BALB/c pneumonic plague model (Figures 3 and 4). There was no significant difference (*P* > 0.02, Wilcoxon test) in the passive protection of the mice from the human sera from three different doses and 35, 196, and 365 days postvaccination.

**Figure 5:**
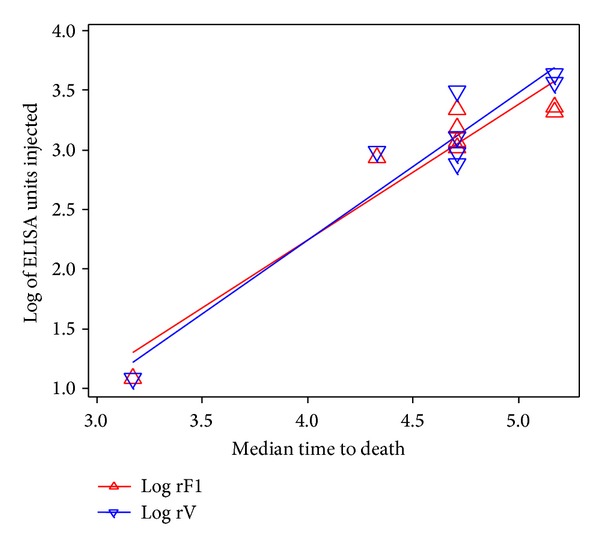
When all of the passive protection data from BALB/c mice were pooled, the log ELISA titre against both rF1 and rV correlated with survival time.

In order to assess the relationship between ELISA titre and MTD, all the data from the different dose experiments were combined. As illustrated in Figure 5, a good correlation was obtained between MTD and the ELISA titre of the sera against both rF1 (*R* ^2^ = 0.91) and rV (*R* ^2^ = 0.92).

The pneumonic plague model was initially developed in BALB/c mice. However, even when using the highest titre human sera available, passive transfer only resulted in a delay in the MTD in BALB/c mice in our hands. We were aware that other published literature had previously described 100% survival in other murine strains [13]. As a result, Hsd:NIHS mice were investigated to determine whether the protection conferred by the human antibodies would be more effective in the different mouse strain.

### 3.4. Aerosol Hsd:NIHS Murine Infection Model

**Figure 6:**
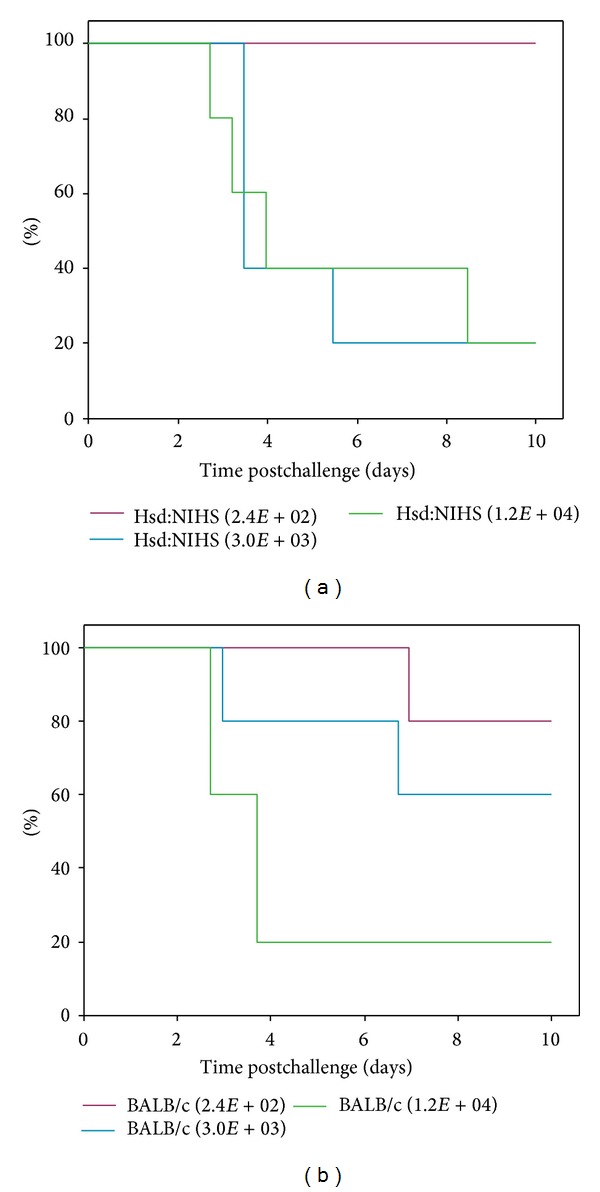
Mouse strain BALB/c and Hsd:NIHS had similar susceptibly to aerosolised* Y. pestis.* (a) shows the survival plots for Hsd:NIHS and (b) shows the survival plots for BALB/c mice. Study (4), *n* = 5 per group. The MTD was calculated using a Kaplan-Meier survival plot.

An aerosol model of murine plague using Hsd:NIHS mice was developed to enable assessment of the efficacy of passive transfer of human antibodies. Results showed that there was no difference in the time to death of Hsd:NIHS mice (*P* > 0.02, Mann-Whitney) compared to BALB/c mice after aerosol challenge of* Y. pestis *(Figure 6), and Table 3 demonstrates that a presented dose of 2.4 LD_50_ or above results in an average MTD of 5.3 days (±0.8 SE).

### 3.5. Comparison of Passive Protection of Human Antibody within Hsd:NIHS and BALB/c Mice

**Figure 7:**
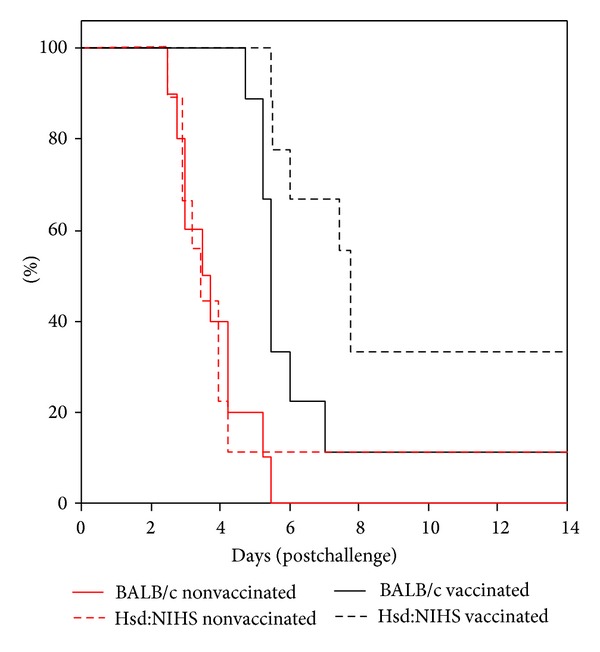
Hsd:NIHS mice had better survival outcomes than BALB/c mice after administration of human serum. Study (5), *n* = 10 mice per group. Mice were injected with 250 *μ*L human serum into the peritoneal cavity, 3–6 hours before being exposed to aerosolised* Y. pestis* using a modified contained Henderson apparatus. The MTD was calculated using a Kaplan-Meier survival plot.

The highest titre human serum available was used to compare the suitability of BALB/c and Hsd:NIHS mouse strains for the assessment of passive protection against pneumonic plague. After exposure to 10.6 LD_50_ of* Y. pestis*, the MTD of BALB/c and Hsd:NIHS with negative control sera was the same at 3.5 days (Figure 7). However, after intraperitoneal administration of high titre human vaccine serum, there were statistically significant (*P* < 0.02, Wilcoxon test) delays in the MTD in both BALB/c and Hsd:NIHS mice of 5.5 to 7.7 days, respectively (Table 3).

### 3.6. Use of Cynomolgus Macaque Serum to Provide Passive Protection

#### 3.6.1. Cynomolgus Macaque Immunisation

**Figure 8:**
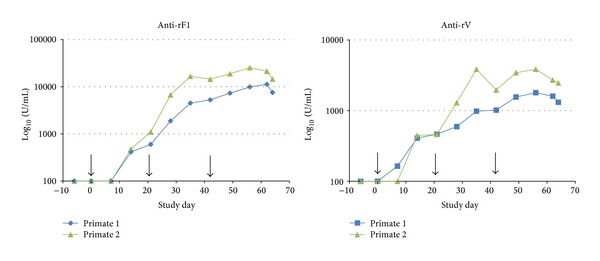
Assessment of anti-rF1 and anti-rV antibody titres in NHP serum after 3 intramuscular immunizations of 10 *μ*g of rF1 and rV antigen. Detectable amounts of anti-rF1 and anti-rV antibodies were found in the serum 2 weeks after the primary immunization, with anti-rF1 consistently having a higher titre.

To confirm that cynomolgus macaque serum can provide a significant level of protection against pneumonic plague, two cynomolgus macaques were immunised intramuscularly with three 10 *μ*g doses of rF1 and rV vaccine 21 days apart. The antibody titres were monitored and the study terminated after the peak of the anti-rF1 and anti-rV antibody responses. The serum isolated at termination was pooled and the anti-rF1 and anti-rV antibody titres were assessed by ELISA (Figure 8).

#### 3.6.2. Pharmacokinetics of Primate Antibody in Mice

To maximise the efficacy of passively transferred antibodies, we examined the pharmacokinetics of cynomolgus macaque serum in the circulating blood stream of Hsd:NIHS mice (Figure 1). The results showed that optimal primate antibody concentrations were reached between three and six hours after intraperitoneal administration of 250 *μ*L. This had a similar profile to human antibodies in BALB/c mice.

#### 3.6.3. Passive Protection with Primate Sera

**Figure 9:**
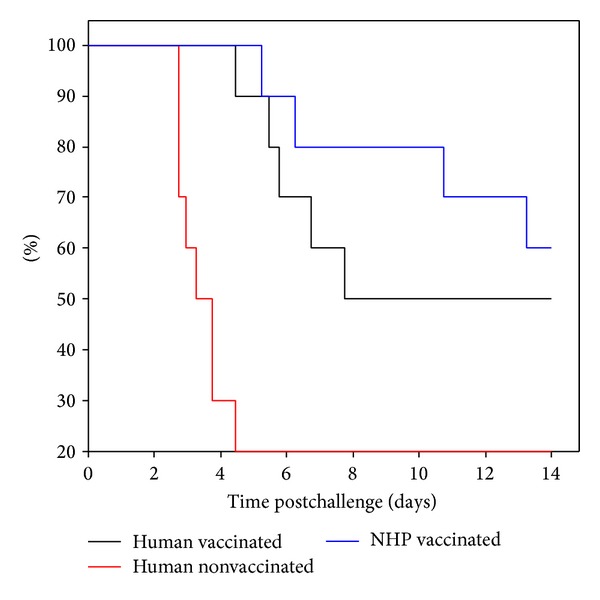
Passive immune therapy with NHP serum provided better protection in Hsd:NIHS mice than human serum after a lethal challenge of* Y. pestis. *Study (6), *n* = 10 mice per group. Mice were administered 250 *μ*L serum into the peritoneal cavity, 3–6 hours before being exposed to aerosolised* Y. pestis* using a modified contained Henderson apparatus. The MTD was calculated using a Kaplan-Meier survival plot.

The cynomolgus macaque serum was used in an assessment of passive protection in Hsd:NIHS mice against an aerosolised challenge of* Y. pestis*. The MTD of the untreated mice was observed to be 3.5 days, whereas the MTD of mice treated with the highest titre of human serum was 7.7 days (Figure 9). The MTD for the mice treated with the cynomolgus macaque serum could not be determined because 60% of the mice survived until day 14. However, there was no statistical difference (*P* > 0.02, Wilcoxon) between the passive protective effect of human and cynomolgus macaque vaccine sera in Hsd:NIHS mice.

## 4. Discussion

The rF1 and rV vaccine was developed to protect against pneumonic plague in humans. In order to enable the licensure of the plague vaccine, information regarding efficacy will need to be provided using animals and the FDAs “animal rule” [32]. In addition, linkage between the human disease and animal studies is essential. An assessment of the protective effect following the passive transfer of antibodies from human vaccines to mice may provide this link.

A model of pneumonic plague was successfully set up by challenging BALB/c mice with aerosolised* Y. pestis* (CO92). This system was both robust and reproducible. To determine whether the antibody response raised against the rF1 and rV plague vaccine was sufficient to protect against pneumonic plague, a passive transfer model in BALB/c mice using human serum was developed. Serum from rF1 and rV vaccines provided significant protection in the pneumonic plague mouse model, as defined by a delay in MTD of between 1 and 2 days. The protective effect of human vaccine sera in mice was found to extend up to 365 days postimmunisation. In addition, this protective effect was not found to be dose dependent as no difference was observed between sera from vaccines that received 40, 80, or 120 *μ*g.

By examining the relationship between human IgG ELISA titre to either rF1 or rV and the MTD, we have demonstrated that the ELISA titres are proportional to the extent of the protective effect. This supports the findings by Fellows et al. [13, 32] which used the recombinant rF1V vaccine and Green et al. [33] who used the rF1 and rV vaccine used within this paper. However, in contrast with the findings of Fellows et al., [13] the passive transfer of human antibody was not providing complete protection as expected. Therefore, we used a mouse strain more closely related to that used by Fellows et al., the outbred Hsd:NIHS strain, and compared them to the inbred BALB/c mouse strain which was used in our earlier studies.

When we compared the susceptibility of Hsd:NIHS and BALB/c mice to aerosolised* Y. pestis* CO92, we found no statistical differences in the mean time to death. To determine whether the antibody response raised against the rF1 and rV plague vaccine was sufficient to protect against pneumonic plague, a passive transfer model in Hsd:NIHS mice using human serum was developed. This was compared to the BALB/c model. Serum from human rF1 and rV vaccines provided significant protection in both the BALB/c and Hsd:NIHS mice strains, as defined by a delay in MTD of between 2 and 4 days, respectively. Restrictions in the quantity of human vaccine trial serum available prevented a repeat pharmacokinetic study of human antibody in Hsd:NIHS mice. The outcome of the pharmacological analysis of cynomolgus macaque antibodies in Hsd:NIHS mice did, however, provide sufficient similarity in the clearance rate of primate antibody for us to be confident that the delay between passive antibody administration and infection was optimal in both murine species.

Cynomolgus macaque sera were also used to assess protection in Hsd:NIHS mice against an aerosolised challenge of* Y. pestis*. The MTD of untreated mice was 3.5 days, whereas those treated with the highest titre of human serum was 7.7. We were unable to provide a median time to death for the NHP serum, as 60% of the mice survived until the end of the experiment.

The passive transfer data presented in this paper supports the findings of previous passive transfer studies from plague vaccine studies [13, 25, 34]. However an interesting result from this study was the difference between the ability of the outbred Hsd:NIHS and the inbred BALB/c to utilise human serum to protect against pneumonic plague. The duration of protection with the human serum was significantly longer in Hsd:NIHS mice than in BALB/c mice. The reason for this difference in protection is unknown and could have important implications when evaluating the efficacy of human antibodies in other passive transfer experiments and should be investigated further. New murine IgG Fc receptors are continually being discovered [35] and are currently being evaluated for their specificity to human IgG. The possibility that Hsd:NIHS mice are able to bind human IgG more effectively than BALB/c mice should be further investigated.

In summary, the results presented in this study demonstrate that passive transfer of human and cynomolgus macaque antibodies raised in response to vaccination with rF1 and rV provides protection in the form of delay in the median time to death in murine pneumonic plague. The data indicate that increasing levels of antibodies result in an increase in the MTD. Our data also demonstrates a good correlation between IgG ELISA titres to rF1 and rV with biological protection. In addition, human serum was better utilized for protection in Hsd:NIHS mice than in BALB/c mice.

## Figures and Tables

**Table 1:** Genetic primer targets of the PCR test used to ensure integrity of the inoculum. All primer probe design was based on GenBank accession number NC_003143 (*Yersinia pestis* CO92, complete genome). MGB is *minor groove binder/nonfluorescent quencher* covalently bound to the 3′ end of the probe. All probes have a 5′ fluorescein (FAM) label.

Gene	Sequence (5′→3′)
HmsF (chromosomal *pgm* locus)	
Forward	CGGAGAAGCCAACGTTCGT
Reverse	TCTTTCACTTTGCGGCAATG
Probe (MGB)	CCGCCTGCACAACG
F1 (pMT1)	
Forward	TTGGCGGCTATAAAACAGGAA
Reverse	CACCCGCGGCATCTGTA
Probe (MGB)	CACTAGCACATCTGTTAAC
Pst (pPCP1)	
Forward	CGGCAATCGTTCCCTCAA
Reverse	GGTCAGGAAAAAGACGGTGTGA
Probe (MGB)	AACCATGACACGGTAGACT
VAg (pCD1)	
Forward	CGGCGGTTAAAGAGAAATGC
Reverse	CATCGCCGAATACACAATGG
Probe (MGB)	TACTGCCATGAACGCC

**Table 2:** A tabular summary of the murine data for each of the challenge experiments described.

Study	Number of mice per group	Minimum body weight (g)	Sex of mice	Source of mice	Strain of mice
Study 1	6	17.7	Female	Harlan	BALB/c
Study 2	10	17.5	Female	Charles Rivers	BALB/c
Study 3	10	17.1	Female	Charles Rivers	BALB/c
Study 4	5	18.0	Female Male	Harlan	BALB/c Hsd:NIHS
Study 5	10	18.0	Female	Harlan	BALB/c Hsd:NIHS
Study 6	10	18.0	Female	Harlan	Hsd:NIHS

**Table 3:** A tabular summary of presented dose of aerosolised *Y. pestis*, passive immune therapy treatment, and MTD in BALB/c and Hsd:NIHS mice. The median time to death (MTD) was calculated using a Kaplan-Meier survival plot. N/D: not determined; over 50% of the mice survived to the end of these experiments; therefore an MTD could not be calculated.

Presented dose		Mouse strain	Study	Passive immune treatment	MTD
(CFU)	LD_50_				
240	0.05	BALB/c	4	No treatment	N/D
3,000	0.6	BALB/c	4	No treatment	N/D
12,000	2.4	BALB/c	4	No treatment	3.7
62,026	12.4	BALB/c	1	Human reference serum	4.92
57,746	11.5	BALB/c	2	Human reference serum	4.18
69,390	13.9	BALB/c	3	Human reference serum	4.71
62,026	12.4	BALB/c	1	Human control negative serum	3.42
57,746	11.5	BALB/c	2	Human control negative serum	2.94
69,390	13.9	BALB/c	3	Human control negative serum	3.17
57,746	11.5	BALB/c	2	D35 40 *μ*g	3.94
57,746	11.5	BALB/c	2	D35 80 *μ*g	4.18
57,746	11.5	BALB/c	2	D35 120 *μ*g	4.18
69,390	13.9	BALB/c	3	D196 40 *μ*g	5.17
69,390	13.9	BALB/c	3	D196 80 *μ*g	5.17
69,390	13.9	BALB/c	3	D196 120 *μ*g	4.71
69,390	13.9	BALB/c	3	D365 40 *μ*g	4.33
69,390	13.9	BALB/c	3	D365 80 *μ*g	4.71
69,390	13.9	BALB/c	3	D365 120 *μ*g	4.71
240	0.05	Hsd:NIHS	4	No treatment	N/D
3,000	0.6	Hsd:NIHS	4	No treatment	3.46
12,000	2.4	Hsd:NIHS	4	No treatment	3.96
53,000	10.6	BALB/c	5	Human vaccine serum	5.48
53,000	10.6	BALB/c	5	Human control negative serum	3.48
53,000	10.6	Hsd:NIHS	5	Human vaccine serum	7.73
53,000	10.6	Hsd:NIHS	5	Human control negative serum	3.48
62,000	12	Hsd:NIHS	6	Human vaccine serum	7.75
62,000	12	Hsd:NIHS	6	Human control negative serum	3.25
62,000	12	Hsd:NIHS	6	Cynomolgus macaque vaccinated serum	N/D
